# Toll-Like Receptors in Atherosclerosis

**DOI:** 10.3390/ijms140714008

**Published:** 2013-07-04

**Authors:** Mika Falck-Hansen, Christina Kassiteridi, Claudia Monaco

**Affiliations:** Kennedy Institute of Rheumatology, NDORMS, University of Oxford, Roosevelt Drive, Headington, Oxford OX3 7FY, UK; E-Mails: m.falck-hansen09@imperial.ac.uk (M.F.-H.); christina.kassiteridi@wolfson.ox.ac.uk (C.K.)

**Keywords:** pattern recognition, inflammation, toll-like receptors, innate immunity, atherosclerosis, cardiovascular disease, macrophages, dendritic cells

## Abstract

Atherosclerosis, the leading cause of cardiovascular disease (CVD), is driven by inflammation. Increasing evidence suggests that toll-like receptors (TLRs) are key orchestrators of the atherosclerotic disease process. Interestingly, a distinct picture is being revealed for individual receptors in atherosclerosis. TLRs exhibit a complex nature enabling the detection of multiple motifs named danger-associated molecular patterns (DAMPs) and pathogen-associated molecular patterns (PAMPs). Activation of these receptors triggers an intracellular signalling cascade mediated through MyD88 or TRIF, leading to the production of pro- and anti-inflammatory cytokines. In this review we explore key novel findings pertaining to TLR signalling in atherosclerosis, including recently described endosomal TLRs and future directions in TLR research.

## 1. Introduction

Atherosclerosis drives CVD, the leading cause of death worldwide [1]. The atherosclerotic process, also described as “hardening of the arteries”, is caused by multiple local and systemic risk factors and often presents itself alongside cardiovascular comorbidities such as type 2 diabetes mellitus (T2DM), the metabolic syndrome, dyslipidaemia and hypertension [2]. There is an increasing body of evidence that innate immune mechanisms may initiate and accelerate atherosclerosis [3]. Recent evidence linked innate sensing to downstream molecular signalling cascades in atherosclerosis [4]. In this review, we will cover the biological significance of TLRs as crucial signal transducers that tailor immune responses during atherosclerosis.

## 2. TLRs: Key Pattern Recognition Receptors (PRRs)

The innate immune system maintains surveillance for exogenous pathogens or cell damage by surface-expressed PRRs [5]. PRRs are located on the cell surface and cytoplasm where they detect PAMPs, such as lipopolysaccharide (LPS), released from Gram-negative bacteria or viral RNA [6]. These receptors are also thought to respond to DAMPs, such as high-mobility group protein B1 (HMGB1), secreted from immune cells when exposed to pro-inflammatory signals [7]. The PRR families consist of TLRs, retinoic acid-inducible gene 1 (RIG-I)-like receptors (RLRs), nucleotide-binding oligomerization domain (NOD)-like receptors (NLRs) and C-type lectin receptors (CLRs) [8] (see Table 1 for a list of endogenous and exogenous TLR ligands).

TLRs are the most well characterised PRRs, of which 11 have been identified in humans and 13 in the mouse [50]. TLRs are expressed by a number of immune cells such as macrophages, dendritic cells (DCs) and non-immune cells, such as epithelial cells [50]. TLR1, TLR2, TLR4, TLR5, TLR6 and TLR11 are expressed in the extracellular space and detect lipopeptides [51]. Bacterial and viral nucleic acids are recognized by TLR3, TLR7, TLR8, TLR9 and the murine TLR13. They are all found on the endoplasmic reticulum, endosomes and lysosomes.

**Figure 1:**
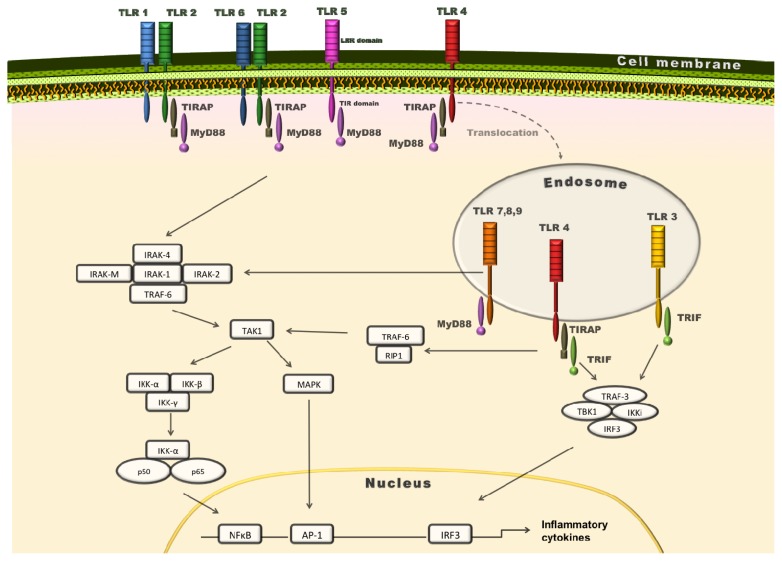
TLR signalling pathways. MyD88 is an adaptor protein, critical in mediating the signalling of all TLRs except TLR3. TIRAP/MAL participates in TLR2 and TLR4 MyD88-mediated signal transduction, in that its *C*-terminus TIRAP/MAL-TIR bridges TLR2 and TLR4 with MyD88 [56,57]. In addition to a TIR-domain, MyD88 harbours a death domain (DD). Upon PAMP recognition by TLRs, its DD interacts with the DD of a member of the IL-1 receptor-associated kinase (IRAK) family, IRAK-4, which consists of a DD and a kinase-like domain. The formation of the MyD88-IRAK-4 complex recruits IRAK-1 and IRAK-2, bringing their kinase-like domains close, resulting in phosphorylation of IRAKs and their subsequent activation. Phosphorylated IRAK-1 or IRAK-2 leave the complex and interact with tumour necrosis factor receptor associated factor 6 (TRAF6), an E3 ubiquitin ligase, to generate Lys63-linked polyubiquitination [58,59]. These polyubiquitin chains bind a complex of TGF-β activated kinase-1 (TAK-1) and TAK-1 binding proteins (TAB) 1, 2 and 3—resulting in TAK1 activation. Activated TAK-1 phosphorylates IKKβ. The subsequent activation of the IKK complex, consisting of IKKα, IKKβ, and NEMO/IKKγ, induces phosphorylation of IKKα and MAP kinases, allowing for the activation of transcription factors and production of inflammatory cytokines.

Upon ligand binding TLRs dimerise and most of them form homodimers, with the exception of TLR1/2, TLR2/6 and TLR4/6/CD36 which heterodimerise [33,51] Structurally, TLRs share an extracellular leucine-rich repeat domain (LRR), responsible for PAMP detection, and a Toll/Interleukin-1 receptor (TIR) domain. The latter dictates TLR-mediated signal transduction. Upon TLR ligand stimulation, the TIR-domain binds to TIR-domain adaptor proteins: myeloid differentiation primary-response protein 88 (MyD88), TIR domain containing adaptor protein (TIRAP) (also known as MyD88-adaptor-like or MAL) and TIR domain-containing adaptor inducing IFN-β (TRIF), the TRIF-related adaptor molecule (TRAM) resulting in two distinct signalling pathways: The MyD88-dependent and the MyD88-independent/TRIF-dependent pathways (Figure 1) [6,52].

## 3. Role of MyD88 in Atherosclerosis

The adaptor protein MyD88 is critical in channelling the signalling of all TLRs, except TLR3 (an illustration of MyD88 signalling is shown in Figure 1). An increasing body of evidence suggests that MyD88, a key protein for TLR signalling, plays a role in disease processes. Its function is illustrated by the effect of knocking out the MyD88 gene in atherosclerosis.

Bjorkbacka *et al.* studied MyD88 deficiency and showed that inactivation of MyD88 led to a reduction in atherosclerosis mediated by reduced macrophage recruitment to the artery wall, associated with reduced chemokine levels [53]. Another study by Michelsen *et al.* showed that *ApoE*^−/−^*MyD88*^−/−^ mice display a diminished aortic atherosclerosis and reduced macrophage accumulation compared to *ApoE*^−/−^ mice [54], indicative of the systemic effect this signalling pathway plays in atherosclerosis disease. However, not all studies have found that MyD88 is detrimental. Recently, key findings suggest that MyD88 plays a role in CD11c^+^ DCs. Subramanian *et al.* focused on the role of CD11c^+^ DC expansion during atherosclerosis [55]. DCs are important sensors of DAMPs and PAMPS. Armed with a plethora of TLRs on their elongated dendrite surface, they are able to sense and dictate adaptive immune responses. Using a bone marrow chimera, where MyD88 is selectively deficient in CD11c^+^ DCs, Subramanian *et al.* transplanted bone marrow into western-diet fed low-density lipoprotein deficient (*LDLR*^−/−^) recipient mice [55]. As a result they observed that recipients had decreased presence of regulatory T cells (Tregs) in the atherosclerotic lesions. The underlying mechanism whereby MyD88-mediated DC activation provides atheroprotection is by promoting Treg generation. Tregs, in turn, abolish T effector cells, inflammatory macrophages and attenuate monocyte recruitment by suppressing MCP-1 production in a TGF-β dependent manner [55]. The blockage of MyD88 is certainly an interesting strategy for the treatment of atherosclerosis, however, due to the broad role of the adaptor molecule in TLR signalling, it could be preferential to target individual TLRs to achieve more specific therapeutic outcomes.

## 4. Functional Diversity of Extracellular TLRs in Atherosclerosis

Both murine and human atherosclerotic lesions display enhanced TLR expression. Studies by Dunzendorfer *et al.* described the effect of disturbed flow on vascular endothelium and demonstrated that endothelial cells subject to conditions of laminar flow *in vitro* were less responsive to TLR2 ligands [60]. In contrast, disturbed flow appeared to induce the same expression and responsiveness as static flow, suggesting that laminar flow reduces the development of TLR2 sensing—in accordance with lesion development sites [60]. In another TLR2 study, reduction in atherogenesis was observed when a complete depletion was obtained in *TLR2*^−/−^*LDLR*^−/−^ crossbred mice compared to *LDLR*^−/−^ mice after 10 and 14 weeks on a high fat diet [61] but not in bone marrow chimera studies, a finding suggestive that selective TLR2 cellular-type expression contributes differentially to lesion development [61]. Mullick *et al.* revealed that TLR2 expression is increased on the surface of endothelial cells at sites prone to development of atherosclerosis, such as the inner curvature of the aortic arch in *LDLR*^−/−^ mice [62]. Work carried out by Madan and Amar revealed that TLR2 mediates diet and/or pathogen associated atherosclerosis. *ApoE*^+/−^*TLR2*^+/+^ mice on a high fat diet and/or bacterial infection exhibited an unstable atherosclerotic plaque phenotype. This was confirmed by the detailed analysis of plaque composition of the proximal aorta, which showed greater macrophage infiltration and apoptosis, diminished smooth muscle cell (SMC) mass, larger lipid core and increased pro-inflammatory cytokine levels compared to *ApoE*^+/≥^*TLR2*^−/−^ and *ApoE*^+/−^*TLR2*^+/−^ controls. Proteomic work on aortic roots of *ApoE*^+/−^*TLR2*^+/+^ mice identified elevated levels of gelsolin, a protein bound to actin, which is suspected to be responsible for increased apoptosis observed in this group [63]. In humans, studies of atheroma cell cultures revealed that blockade of TLR2 and MyD88 inhibits NF-κB activation and matrix metalloproteinase (MMP) production, suggesting that MyD88-mediated TLR2 signalling contributes to human atherosclerosis [64].

Plaque necrosis is known to arise as an effect of macrophage apoptosis in advanced lesions [65]. Recently, Seimon *et al.* described the effect from TLR2 and CD36 detection of oxLDL leading to apoptosis in macrophages undergoing endoplasmic reticulum-induced stress [66], which could explain a mechanism whereby macrophages undergo necrosis and form the plaque necrotic core [66]. Furthermore, work by Higashimori *et al.* found that TLR2 deficiency diminishes foam cell accumulation in lesion-prone areas of the aorta of *ApoE*^−/−^ mice [67] yielding further support to the pathogenic effect of TLR2 in murine models of atherosclerosis by Mullick *et al.* [62]. A deficiency in either TLR6 or TLR1 did not reduce atherosclerosis disease induced by exposure to a high fat diet [68], all together suggesting that TLR6 and TLR1 individually may not be sufficient per se, but will act together with TLR2 as heterodimers [68].

*ApoE*^−/−^ mice deficient in *TLR4* exhibited a decreased development of atherosclerotic lesions (up to 55% less), while at the same time; monocytes infiltrated atherosclerotic lesions to a lesser extent (with a 65% decrease) in *ApoE*^−/−^*TLR4*^−/−^ compared to *ApoE*^−/−^ mice [54,61]. An increase in TLR4 expression was observed in atherosclerosis after oxLDL stimulation [32]. Accordingly, genetic deletion of *TLR4* reduces atherosclerotic lesion development by 24% [54] and macrophage infiltration by 65% accompanied with decreased IL-12 and MCP-1 levels [54]. Seimon *et al.* investigated the role of TLR2 and TLR4 in macrophage apoptosis. In their studies transferring *TLR2*^−/−^*TLR4*^−/−^ donor bone marrow into *LDLR*^−/−^ recipients on a high fat diet, they observed a 45% decrease in necrotic core area when TLR2 and TLR4 were absent in the bone marrow cells in *LDLR*^−/−^ recipients compared to controls [68]. This study suggested an effect of *TLR2*^−/−^*TLR4*^−/−^ on necrosis, but also showed no statistical differences amongst body weight, total plasma cholesterol, HDL and triglycerides, FPLC lipoprotein profile or aortic root lesion area when TLR2 and TLR4 were absent.

In another study by Choi *et al.* TLR4 was found to detect minimally oxidized low-density lipoprotein (mmLDL); to act as a mediator of macropinocytosis and, eventually, to play a role in formation of pathognomonic “foam cells” [69]. Interestingly, TLR4 was found to contribute to foam cell formation to a greater extent than TLR2 [67]. Stewart *et al.* demonstrated that the complex recognising oxLDL is composed of TLR4, TLR6 and the scavenger receptor CD36 presenting itself as a heterodimer promoting sterile inflammation [33]. In contrast to previous studies on TLR2, when *ApoE*^−/−^ mice deficient in TLR4 were infected with *P. gingivalis* they were paradoxically more susceptible for developing atherosclerosis, presenting with increased levels of inflammatory Th17 cells [70].

## 5. Endosomal TLRs: Friends or Foes of the Arterial Wall?

A different story unfolded from TLR3 in atherosclerosis—an endosomal TLR that signals via TRIF and is MyD88-independent. Cole *et al.* described an unexpected protective role for TLR3 in atherosclerosis [71]. Studying human atheroma-derived smooth muscle cells they observed an increased expression of TLR3 and pro- and anti-inflammatory responses to dsRNA *in vitro*. Following on this, *in vivo* neointima formation in their perivascular collar-induced injury model was reduced after administration of the TLR3 synthetic analog polyinosine polycytidylic acid (PolyI:C) in *TLR3*^+/+^*ApoE*^−/−^ mice compared with *TLR3*^−/−^*ApoE*^−/−^ mice. Moreover, Cole *et al.* surprisingly observed that *TLR3*^−/−^*ApoE*^−/−^ mice had earlier atherosclerosis than *TLR3*^+/+^*ApoE*^−/−^ counterparts, suggesting that TLR3 is protective [71]. The TLR3 downstream signalling cascade activates TRIF, recruiting TRAF-3, TBK1, IKKi and IRF3. In a study led by Curtiss *et al.* that examined the TRIF mutated gene (Lps2) in *LDLR*^−/−^ mice, the authors found that *LDLR*^−/−^ mice with lack-of-function mutations in TRIF (Lps2) were significantly protected from atherosclerosis, assessed by heart sinus and aorta lesion size quantifications, where the mice displayed fewer observed lesional macrophages [68]. *TLR3*^−/−^*LDLR*^−/−^ mice were assessed for the same readouts. The data from this study suggests that hyperlipidaemia leading to endogenous activation of TRIF signalling is pro-atherogenic, however, TLR3 deficiency seemed protective as TLR3 knockouts exhibited disease enhancement [72]. In contrast, Zimmer *et al.* demonstrated that TLR3 activation in the endothelium impairs endothelial function—a pro-atherogenic development. Injection of intravenous TLR3 ligand polyI:C impaired endothelium-dependent vasodilation, increased vascular production of reactive oxygen species and produced reduced reendothelialization following carotid injury in WT mice compared to controls [73]. *ApoE*^−/−^*TLR3*^−/−^ mice exhibited improved endothelial function compared with *ApoE*^−/−^*TLR3*^+/+^ mice, unravelling a detrimental role on endothelial function mediated through TLR3 [73]. Moreover, Lundberg *et al.* recently reported a detrimental effect on atherosclerosis pertaining to TLR3 and TLR4 and downstream TRIF and TRAM adaptor signalling in hematopoietic cells [74]. Deleting either *TRAM* or *TRIF* in myeloid cells was sufficient to attenuate vessel inflammation and protect against atherosclerosis, as shown by reduced aortic inflammation. This was indicated by lower aortic levels of pro-inflammatory mediators, and a reduction in T cell influx [74]. Different outcomes from these studies on endosomal TLR signalling warrant continuing research to fully understand the mechanisms underlying MyD88-independent TRIF signalling in the pathophysiology of atherosclerosis. It is currently unclear whether the opposing outcomes emerged in these studies are explained by the different experimental conditions (e.g., use of high fat or chow diets, whole body deficiency or bone marrow chimeras), or if they point to a true duplicitous role of endosomal TLR3 and its associated signalling in atherosclerosis. It is possible to speculate that the outcome of TLR3 activation may be different when it involves myeloid cells (detrimental) or other cells (protective). Further targeted deletion studies will be needed to address this important point.

The endosomal receptors TLR7 and TLR8 detect viral ssRNA and self-RNA released from necrotic cells [22]. Some studies have suggested that TLR8 lacks signalling function in mice, whereas TLR7 has been determined as functional [75]. In humans, both TLR7 and TLR8 signal and activate gene transcription [76]. In their study on TLR7 in atherosclerosis, Salagianni *et al.* found that TLR7 could play a protective role by constraining monocyte/macrophage pro-inflammatory activity [77]. The authors showed that *TLR7*^−/−^*ApoE*^−/−^ mice displayed elevated levels of necrotic core formation, lipid deposition, macrophage infiltration, and pro-inflammatory cytokine production, and reduced presence of SMCs and collagen. It was suggested that TLR7 hinders the expression of inflammatory Ly6C^hi^ monocytes and inflammatory M1 macrophages, a MCP-1-mediated process, possibly triggered by the pathogenic PRRs TLR2 and TLR4 [77]. Signalling-dependent IFN responses could explain the distinct roles of TLRs in atherosclerosis. For example, the receptors TLR2, TLR7 and TLR9 all signal via the MyD88-pathway inducing downstream activation of NF-κB signalling [78]. Yet, TLR7 and TLR9 have the specific ability to induce high amounts of type I IFN production. It is unclear if this can really explain the beneficial outcome of TLR7 signalling, since bone marrow chimeras for IFN-β exhibit reduced atherosclerosis [79], indicating that other downstream mechanisms could modulate the beneficial effect of TLR7.

## 6. TLRs in Cardiovascular Risk Factors

TLRs have also been implicated to play a role in the pathogenesis of risk factors. The first link to be identified in this direction was with the metabolic syndrome. In a study led by Vijay-Kumar, the authors demonstrated that transplantation of gut microbiota from *TLR5*^−/−^ mice to germ-free WT mice generated weight gain in recipients, including changes of metabolic syndrome [80] indicating that a malfunctioning of TLRs and the innate immune system may drive insulin resistance. An increase in the presence of Firmicutes and Actinobacteria and reduction in Bacteroidetes in the gut causes LPS leakage into the circulation activating TLR4 and leads to impaired insulin signalling [81]. Saturated fatty acids stimulate TLR2, promoting insulin resistance through the unfolded protein response (UPR) and nitric oxide (NO) production [82], demonstrating a connection between TLR2 and obesity.

In particular, animal models have been proved valuable for linking TLR3, TLR4, TLR7/8 and TLR9 activation with vascular hypertension. Tinsley *et al.* demonstrated that pregnant rats treated with poly:IC exhibited preeclampsia, including high systolic blood pressure, endothelial dysfunction, proteinuria and increased foetal malformation [83]. The same preeclampsia-symptoms, in addition to excessive inflammation, were shown in pregnant mice, after TLR3/7/8 activation following treatment with polyI:C, R-837, and CLO97 agonists, respectively. The same authors examined TLR3/7/8 activation in placentas from women diagnosed with preeclampsia and found increased mRNA levels of TLR3/7/8 but also elevated proinflammatory cytokine levels. However, further studies are needed to show whether TLR activation occurs before or after preeclampsia progression [84]. Additionally, during pregnancy, TLR9 activation elicits features of preeclampsia in rats. It has been speculated that this occurs due to mtDNA release from necrotic cells, which activates TLR9, and thus ERK1/2-dependent signalling, resulting in vascular contractility. Another proposal is that TLR9 activation elicits proinflammatory responses, leading to systemic maternal inflammation [85]. Interestingly, anti-TLR4 treatment has been shown to be beneficial in spontaneously hypertensive rats by decreasing blood pressure and vascular contractility but also by attenuating the expression of pro-inflammatory cytokines, including COX-2 and IL-6 [86]. Furthermore, TLR2 deficiency in mice fails to increase arterial blood pressure after administration of HDL from children and adults with chronic kidney dysfunction (HDL^CKD^), suggesting that TLR2 activation is involved in HDL-mediated endothelial dysfunction and hypertension [87].

Importantly, T2DM patients have significantly elevated TLR2 and TLR4 expression [88]. LPS treatment of abdominal subcutaneous adipocytes from T2DM patients, results in increased TLR2 expression, which could be a possible underlying mechanism of inflammation in T2DM [89]. Furthermore, smoke-derived oxidants are able to activate TLR2 signalling [90].

In conclusion, there is ample scope for further dissecting the requirement of TLRs in a variety of CVD-associated comorbidities. Future research will need to gain mechanistic insight on the intricate balance of direct and risk factor-mediated effects of TLRs in CVD.

## 7. Promising Perspectives for Treating TLRs in Atherosclerosis

Functional analysis of human carotid endarterectomies has revealed that TLR2 blockade can exert beneficial effects mediated by inhibition of the production of pro-inflammatory cytokines, chemokines and MMPs and by attenuating NF-κB activity [64]. Accordingly, decreasing TLR2 expression via atorvastatin treatment exhibited anti-atherosclerotic effects in human arterial endothelial cells mediated by reduced susceptibility to MALP-2 activation [91]. In 2010, Arslan *et al.* demonstrated that a monoclonal antibody against TLR2 (OPN-301) results in reduced neutrophil, macrophage, and T-lymphocyte infiltration, and reduced the production of proinflammatory TNF-α, IL-1α and GM-CSF in the mouse model [92]. Later in 2012, Arslan *et al.* reported the first humanized anti-TLR2 antibody, OPN-305, which reduced infarct size, preserved systolic function and eventually prevented myocardial damage in a pig model of Ischemia/Reperfusion Injury [93,94]. DPP-4 (CD26) inhibitor alogliptin reduced atherosclerotic lesion size in diabetic mice and inhibited TLR4-mediated pro-inflammatory cytokine expression *in vitro* [95]. Additionally, *R. sphaeroides* LPS (Rs-LPS) has been utilized as a TLR4 antagonist, which prevented the expression of pro-atherogenic factors IL-6 and MMP-9, macrophage accumulation in atherosclerotic plaques and NF-κB activity in 14-week old *ApoE*^−/−^ mice [96]. The therapeutic options existing so far to target TLRs are quite limited and further preclinical development is needed. The evidence for TLR2 is somewhat stronger due to the existence of a powerful blocking antibody. As new tools are developed to block the other relevant TLRs, more evidence will be acquired on the feasibility and efficacy of their blockade in CVD.

## 8. Conclusions

TLRs are key orchestrators of the early innate immune defence mechanisms by activating canonical and non-canonical pathways of inflammation. During atherosclerosis, these mechanisms can be detrimental, as seen by the activation of TLR2 and TLR4. Detection of oxLDL mediated by complexes formed from TLR4 and TLR6 in partnership with scavenger receptor CD36 [33] has revealed that TLRs are able to sense pro-atherogenic stimuli giving rise to a danger-sensing pattern that was previously unknown. TLR2 presents as a promising target given its proven efficacy in recent preclinical studies [92,94]. There is also growing support for TLR4, although developing blockers for this TLR has proven rather complex. The current state of the art suggests that blocking TLR2 and perhaps TLR4 may reduce lesion formation and inflammation, while TLR2 blockade also reduces infarct size. The effect on plaque rupture has not been demonstrated, yet the mechanism of action of TLR2 is clearly the reduction of inflammation and MMP production [64] supporting a key role for TLR2 induced inflammation in altering plaque stability.

Early studies on TLR signalling in atherosclerosis have recently been supplemented by surprising effects exerted on arterial lesions, such as those mediated by the endosomal TLR3 and TLR7 receptors that are able to detect dsRNA and ssRNA, respectively. New developments in the basic understanding of the roles of TLR3, TLR6 and TLR7 from *in vitro* studies and pre-clinical models of disease may open up new avenues of TLR-targeted therapy for atherosclerosis. Further studies on the contribution of other endosomal pattern recognition pathways, including those mediated by RLRs and NLRs may provide alternative approaches, furthering the understanding of the complexity of atherosclerosis and will allow for novel strategies to target this disease in years to come.

## Figures and Tables

**Table 1:** TLRs with respective exogenous and endogenous ligands.

TLR receptor	Exogenous ligands	Endogenous ligands	Exogenous source
TLR1	Tri-acyl lipopeptides [9]	Not determined	Mycoplasma
TLR1/2	Soluble factors [10]; PAM3	Not determined	Gram-negative bacteria (*Neisseria meningitidis*)
TLR2	Peptidoglycan [11]; Glycoinositolphospholipids; Glycolipids; Porins; Zymosan; Atypical LPS [12–14]	HMGB1 [15–17]; oxLDL [18]; Serum Amyloid A [19]; Amyloid beta [20]	Gram positive bacteria; *Trypanozoma cruzi; Treponema maltophilum;* Fungi; *Leptospira interrogans; Porphyromonas gingivalis*
TLR2/6	Lipoproteins [21]; Zymosan; Lipoteichoic acids [22]; FSL-1 [23]	Heat-shock proteins such as HSP60 and 70 [24] Versican [25]	Gram positive Bacteria; Fungi
TLR3	dsRNA; (PolyI:C) [26]	mRNA[27]	Virus
TLR4	Lipopolysaccharide [28]; Glycoproteins [29]; Taxol, RSV Fusion Protein [12]; HSP60 [30]	Tenascin C [31]; oxLDL [32]; Amyloid beta [33]; HSP22 [34]; HSP70 [35]; HSP72 [36]; Gp96 [37]; ECM fragments [38]; Oxidized phospholipids [39]; Betadefensin-2 [40]; HMGB1 [15–17]	Virus; plant; *Chlamydia pneumoniae*
TLR5	Flagellin [41]	Not determined	Bacteria
TLR6	Di-acyl lipopeptides [42]	Not determined	Mycoplasma
TLR7	Imidazoquinolines; loxoribine and Bropirine [12]; ssRNA [22]	ssRNA (immune complex) [22]	Virus
TLR7/8	ssRNA; Imidazoquinolines [43]	ssRNA (immune complex) [22]	Virus
TLR7/9	Not determined	Nucleic acid-containing immune complexes [44–47]	Not determined
TLR9	CpG-DNA [48]; CpG oligonucleotide; Hemozoin [49]	Chromatin IgG complex [44]	Bacteria; Parasites (*Plasmodium*)
